# *SNeP*: a tool to estimate trends in recent effective population size trajectories using genome-wide SNP data

**DOI:** 10.3389/fgene.2015.00109

**Published:** 2015-03-20

**Authors:** Mario Barbato, Pablo Orozco-terWengel, Miika Tapio, Michael W. Bruford

**Affiliations:** ^1^School of Biosciences, Cardiff UniversityCardiff, UK; ^2^MTT Agrifood Research Finland, Biotechnology and Food ResearchJokioinen, Finland

**Keywords:** effective population size, linkage disequilibrium, SNPChip, demography, large scale genotyping

## Abstract

Effective population size (*N_e_*) is a key population genetic parameter that describes the amount of genetic drift in a population. Estimating *N_e_* has been subject to much research over the last 80 years. Methods to estimate *N_e_* from linkage disequilibrium (LD) were developed ~40 years ago but depend on the availability of large amounts of genetic marker data that only the most recent advances in DNA technology have made available. Here we introduce *SNeP*, a multithreaded tool to perform the estimate of *N_e_* using LD using the standard PLINK input file format (.ped and.map files) or by using LD values calculated using other software. Through *SNeP* the user can apply several corrections to take account of sample size, mutation, phasing, and recombination rate. Each variable involved in the computation such as the binning parameters or the chromosomes to include in the analysis can be modified. When applied to published datasets, *SNeP* produced results closely comparable with those obtained in the original studies. The use of *SNeP* to estimate *N_e_* trends can improve understanding of population demography in the recent past, provided a sufficient number of SNPs and their physical position in the genome are available. Binaries for the most common operating systems are available at https://sourceforge.net/projects/snepnetrends/.

## Introduction

Effective population size (*N_e_*) is an important genetic parameter that estimates the amount of genetic drift in a population, and has been described as the size of an idealized Wright–Fisher population expected to yield the same value of a given genetic parameter as in the population under study (Crow and Kimura, 1970). *N_e_* sizes can be influenced by fluctuations in census population size (*N_c_*), by the breeding sex ratio and the variance in reproductive success.

*N_e_* estimation can be achieved using approaches that fall into three methodological categories: demographic, pedigree-based, or marker-based (Flury et al., 2010). Pedigree data have been traditionally used to obtain *N_e_* estimates in livestock. However, reliable estimates of *N_e_* depend on the pedigree being complete. This state of knowledge is feasible in some domestic populations, the demographic parameters of which have been accurately monitored for a sufficiently large number of generations. However, in practice, the applicability of this approach remains limited to a few cases involving highly managed breeds (Flury et al., 2010; Uimari and Tapio, 2011). One solution to overcome the limitation of an incomplete pedigree is to estimate the recent trend in *N_e_* using genomic data. Several authors have recognized that *N_e_* could be estimated from information on linkage disequilibrium (LD) (Sved, 1971; Hill, 1981). LD describes the non-random association of alleles in different loci as a function of the recombination rate between the physical positions of the loci in the genome. However, LD signatures can also result from demographic processes such as admixture and genetic drift (Wright, 1943; Wang, 2005), or through processes such as “hitchhiking” during selective sweeps (Smith and Haigh, 1974) or background selection (Charlesworth et al., 1997). In such scenarios alleles at different loci become associated independently of their proximity in the genome. Assuming that a population is closed and panmictic, the LD value calculated between neutral unlinked loci depends exclusively on genetic drift (Sved, 1971; Hill, 1981). This occurrence can be used to predict *N_e_* due to the known relationship between the variance in LD (calculated using allele frequencies) and effective population size (Hill, 1981).

Recent advances in genotyping technology (e.g., using SNP bead arrays with tens of thousands of DNA probes) have enabled the collection of vast amounts of genome-wide linkage data ideal for estimating *N_e_* in livestock and humans among others (e.g., Tenesa et al., 2007; de Roos et al., 2008; Corbin et al., 2010; Uimari and Tapio, 2011; Kijas et al., 2012). However, a software tool that enables estimation of *N_e_* from LD is lacking, and researchers currently rely on a combination of tools to manipulate data, infer LD, and tend to use bespoke scripts to perform the appropriate calculations and estimate *N_e_*.

Here we describe *SNeP*, a software tool that allows the estimation of *N_e_* trends across generation using SNP data that corrects for sample size, phasing and recombination rate.

## Materials and methods

The method *SNeP* uses to calculate LD depends on the availability of phased data. When the phase is known the user can select Hill and Robertson (1968) squared correlation coefficient that makes use of haplotype frequencies to define LD between each pair of loci (Equation 1). However, in the absence of a known phase, squared Pearson's product-moment correlation coefficient between pairs of loci can be selected. While these two approaches are not the same, they are highly comparable (McEvoy et al., 2011):

(1) $$r^{2}\;=\frac{{\left(p_{AB}-p_{A}p_{B}\right)}^{2}}{p_{A}\left(1-p_{A}\right)p_{B}\left(1-p_{B}\right)}$$

(2) $$r_{X,Y}^{2}=\frac{{\left[\sum_{i\;=\;1}^{n}\left(X_{i}-\bar{X}\right)\left(Y_{i}-\bar{Y}\right)\right]}^{2}}{\sum_{i\;=\;1}^{n}{\left(X_{i}-\bar{X}\right)}^{2}\sum_{i\;=\;1}^{n}{\left(Y_{i}-\bar{Y}\right)}^{2}}$$

where *p_A_* and *p_B_* are respectively the frequencies of alleles A and B at two separate loci (*X*, *Y*) measured for *n* individuals, *p_AB_* is the frequency of the haplotype with alleles A and B in the population studied, *X* and *Y* are the mean genotype frequencies for the first and second locus respectively, *X_i_* is the genotype of individual *i* at the first locus and *Y_i_* is the genotype of individual *i* at the second locus. Equation (2) correlates the genotypic allele counts instead of the haplotype frequencies and is not influenced by double heterozygotes (this approach results in the same estimates as the --r2 option in PLINK).

*SNeP* estimates the historic effective population size based on the relationship between *r*^2^, *N*_*e*_, and *c* (recombination rate), (Equation 3—Sved, 1971), and enabling users to include corrections for sample size and uncertainty of the gametic phase (Equation 4—Weir and Hill, 1980):

(3) $$E\left(r^{2}\right)={\left(1+4N_{e}c\right)}^{-1}$$

(4) $$r_{adj}^{2}=r^{2}-{\left(\beta n\right)}^{-1}$$

*w*here *n* is the number of individual sampled, β = 2 when the gametic phase is known and β = 1 if instead the phase is not known.

Several approximations are used to infer the recombination rate using the physical distance (δ) between two loci as a reference and translating it into linkage distance (*d*), which is usually described as *Mb*(δ) ≈ *cM*(*d*). For small values of *d* the latter approximation is valid, but for larger values of *d* the probability of multiple recombination events and interference increases, moreover the relationship between map distance and recombination rate is not linear, as the maximum recombination rate possible is 0.5. Thus, unless using very short δ, the approximation *d* ≈ *c* is not ideal (Corbin et al., 2012). We therefore implemented mapping functions to translate the estimated *d* into *c*, following Haldane (1919), Kosambi (1943), Sved (1971), and Sved and Feldman (1973). Initially *SNeP* infers *d* for each pair of SNPs as directly proportional to δ according to *d* = *k*δ where *k* is a user defined recombination rate value (default value is 10^−8^ as in *Mb* = *cM*). The inferred value of δ can then be subjected to one of the available mapping functions if required by the user.

Solving Equation (3) for *N_e_* and including all the corrections described, allows the prediction of *N_e_* from LD data using (Corbin et al., 2012):

(5) $$N_{T\left(t\right)}={\left(4f\left(c_{t}\right)\right)}^{-1}\left(E{\left[r_{adj}^{2}|c_{t}\right]}^{-1}-\alpha \right)$$

where *N_t_* is the effective population size *t* generations ago calculated as *t* = (2*f*(*c_t_*))^−1^ (Hayes et al., 2003), *c_t_* is the recombination rate defined for a specific physical distance between markers and optionally adjusted with the mapping functions mentioned above, *r*^2^_*adj*_ is the LD value adjusted for sample size and α:= {1, 2, 2.2} is a correction for the occurrence of mutations (Ohta and Kimura, 1971). Therefore, LD over greater recombinant distances is informative on recent *N_e_* while shorter distances provide information on more distant times in the past. A binning system is implemented in order to obtain averaged *r*^2^ values that reflect LD for specific inter-locus distances. The binning system implemented uses the following formula to define the minimum and maximum values for each bin:

(6a) $$b_{i}^{min}=minD+\left(maxD-minD\right){\left(\frac{b_{i}-1}{totBins}\right)}^{x}$$

(6b) $$b_{i}^{max}=minD+\left(maxD-minD\right){\left(\frac{b_{i}}{totBins}\right)}^{x}$$

Where *b_i_* (ℕ^1^) is the *i^th^* bin of the total number of bins (*totBins*), *minD*, and *maxD* are respectively the minimum and the maximum distance between SNPs and *x* is a positive real number (ℝ^0^) When *x* equals 1, the distribution of distances between the bins is linear and each bin has the same distance range. For larger values of *x* the distribution of distances changes allowing a larger range on the last bins and a smaller range on the first bins. Varying this parameter allows the user to have a sufficient number of pairwise comparisons to contribute to the final *N_e_* estimate for each bin.

### Example application

We tested *SNeP* with two published datasets that had been previously used to describe trends in *N_e_* over time using LD, *Bos indicus* [54,436 SNPs of 423 East African Shorthorn Zebu (SHZ)–Mbole-Kariuki et al., 2014, data available at Dryad Digital Repository: doi:10.5061/dryad.bc598.] and *Ovis aries* [49,034 SNPs genotyped in 24 Swiss White Alpine (SWA), 24 Swiss Black-Brown Mountain sheep (SBS), 24 Valais Blacknose sheep (VBS), 23 Valais Red sheep (VRS), 24 Swiss Mirror sheep (SMS) and 24 Bundner Oberländer sheep (BOS)–Burren et al., 2014]. The *r*^2^ estimates for the cattle datasets were obtained by the authors using GenABLE (Aulchenko et al., 2007) using a minimum allele frequency (MAF) < 0.01 and adjusting the recombination rate using Haldane's mapping function (Haldane, 1919). The *r*^2^ estimates of the sheep data were calculated by the authors using PLINK-1.07 (Purcell et al., 2007), with a MAF < 0.05 and no further corrections. For both autosomal datasets *r*^2^ estimates where corrected for sample size using equation (4) with β = 2. For these comparative analyses the *SNeP* command line included the same parameters used for the published data apart from the *r*^2^ estimates, calculated through genotype count and the use of *SNeP*'s novel binning strategy.

## Results

**Figure 1:**
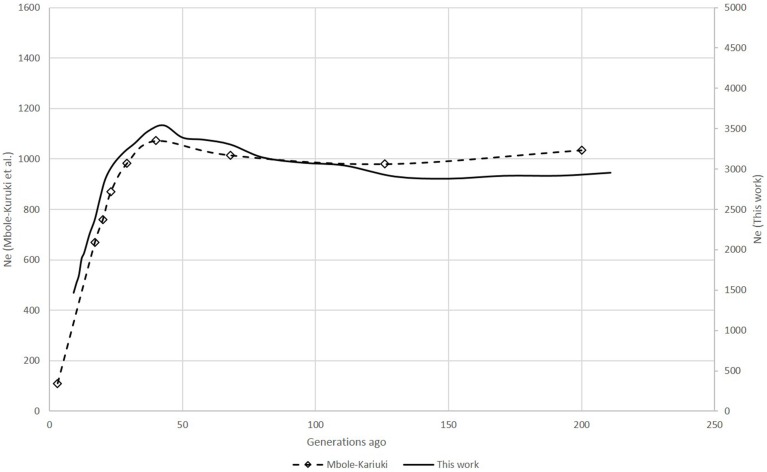
**Comparison of *N_e_* trends of six Swiss sheep breeds according to Burren et al. (2014) (dashed lines) and this work (solid lines)**.

*SNeP* is a multithreaded application developed in C++ and binaries for the most common operating systems (Windows, OSX, and Linux) can be downloaded from https://sourceforge.net/projects/snepnetrends/. The binaries are accompanied by a manual describing the step-by-step use of *SNeP* to infer trends in *N_e_* as described here. *SNeP* produces an output file with tab delimited columns showing the following for each bin that was used to estimate *N_e_*: the number of generations in the past that the bin corresponds to (e.g., 50 generations ago), the corresponding *N_e_* estimate, the average distance between each pair of SNPs in the bin, the average *r*^2^ and the standard deviation of *r*^2^ in the bin, and the number of SNPs used to calculate *r*^2^ in the bin. This file can be easily imported in Microsoft Excel, R or other software to plot the results. The plots shown here (Figures 1, **3**) correspond to the columns of generations ago and *N_e_* from the output file. The column with the *r*^2^ standard deviation is provided for users to inspect the variance in the *N_e_* estimate in each bin, particularly for those bins reflecting older time estimates and which are less reliable as the number of SNPs used to estimate *r*^2^ becomes smaller.

The format required for the input files is the standard PLINK format (ped and map files) (Purcell et al., 2007). *SNeP* allows the users to either calculate LD on the data as described above, or use a custom precalculated LD matrix to estimate *N_e_* using Equation (5).

The software interface allows the user to control all parameters of the analysis, e.g., the distance range between SNPs in bp, and the set of chromosomes used in the analysis (e.g., 20–23). Additionally, *SNeP* includes the option to choose a MAF threshold (default 0.05), as it has been shown that accounting for MAF results in unbiased *r*^2^ estimates irrespective of sample size (Sved et al., 2008). *SNeP*'s multithreaded architecture allows fast computation of large datasets (we tested up to ~100K SNPs for a single chromosome), for example the BOS data described here was analyzed with one processor in 2′43″, the use of two processors reduced the time to 1′43″, four processors reduced the analysis time to 1′05″.

### Zebu example

For the zebu analysis, the shapes of the *N_e_* curves obtained with *SNeP* and their published data trends showed the same trajectory with a smooth decline until around 150 generations ago, followed by an expansion with a peak around 40 generations ago and ending in a steep decline on the most recent generations (Figure 1). However, while the trends in both curves were the same, the two approaches resulted in different *N_e_* estimates, with *SNeP*'s values being approximately three-fold larger than those in the original paper. While we attempted to use the authors' parameters in our analyses, some differences were inevitable, i.e., the original publication of the cattle data estimated *r*^2^ with a different approach to that implemented in *SNeP*. Analyses with *SNeP* were based on genotypes, while the original analysis was based on inferred two locus haplotypes, which results in the published data showing an expected *r*^2^ of 0.32 at the minimum distance, while our estimates was 0.23. Similarly, Mbole-Kariuki et al. (2014) obtained a background level *r*^2^ = 0.013 around 2 Mb, while our estimate at the same distance was 0.0035 (data not shown). Consequently, as our estimates of LD were consistently smaller than Mbole-Kariuki et al. (2014) it is expected that our *N_e_* estimates should be larger. While this observation highlights the importance of a careful choice of the parameters and their thresholds, it is important to highlight that although the absolute magnitude of the *N_e_* values is different, the trends are almost identical.

### Swiss sheep example

**Figure 2:**
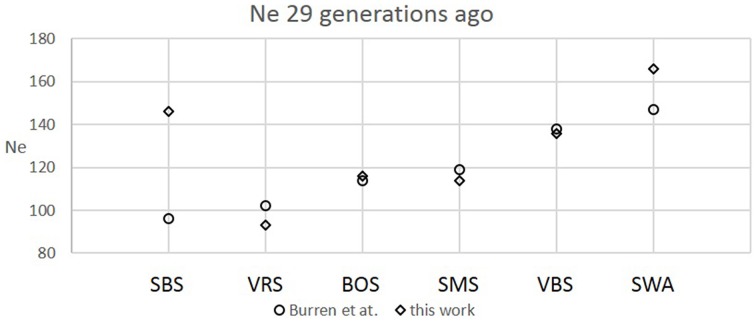
**Comparison between recent *N_e_* values calculated at the 29th generation in this work and Burren et al. (2014) for six Swiss sheep breeds**.

**Figure 3:**
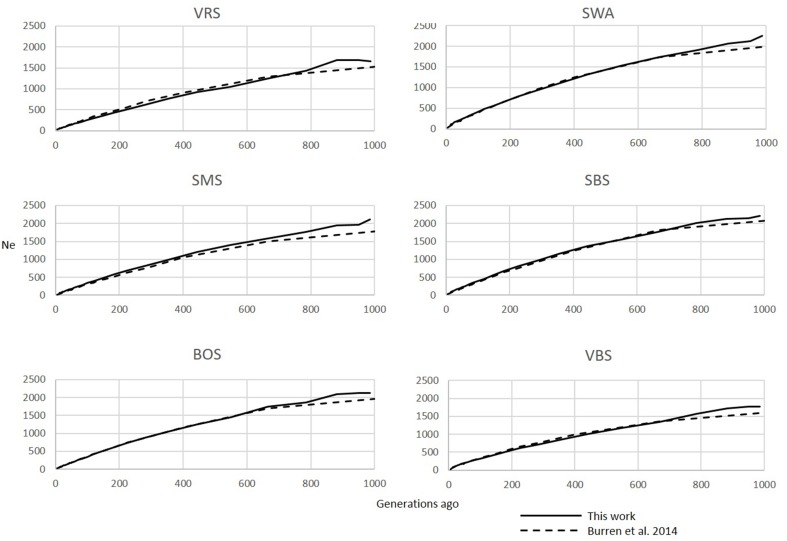
**Comparison of *N_e_* trends for the last 250 generations in the SHZ data obtained by Mbole-Kariuki et al. (2014) (dashed line) and using *SNeP* (solid line)**.

The six Swiss sheep breeds analyzed with *SNeP* produced comparable results with those from the original paper (Figure 2), with mostly overlapping *N_e_* trend curves (Figure 3). However, the general trend in *N_e_* showed a decline toward the present. *SNeP* produced slightly larger values of *N_e_* for the more distant past (700–800 generations). This is due to the different binning system used in *SNeP*, which allows the user to obtain a more even distribution of pairwise comparisons within each bin (i.e., the number of SNP pairwise comparisons within each bin is comparable). For the time span extending beyond 400 generations ago, Burren et al. (2014) used only three bins in their analysis (centered at 400, 667, and 2000 generations ago) while for the same time span *SNeP* used 5 bins with a number of pairwise comparisons dependent to the range defined with formulae 6a,b. Consequently, Burren and colleagues' approach ends with a higher density of data describing the most recent generations than describing the oldest generations. Therefore, the use of fewer bins tends to increase the presence of smaller values of *N_e_* in each bin, consequently lowering the average *N_e_* value for each bin. The *N_e_* values for the recent past, compared at the 29th generation in the past, gave very similar results. The largest difference (50) was obtained for the SBS breed.

## Discussion

Analysis of *N_e_* using LD data was first demonstrated 40 years ago, and has been applied, developed and improved since (Sved, 1971; Hayes et al., 2003; Tenesa et al., 2007; de Roos et al., 2008; Corbin et al., 2012; Sved et al., 2013). The traditionally small number of SNPs analyzed is no longer a limitation, since SNP Chips comprise an extremely large number of SNPs, available in a short time and at a reasonable price. This has boosted the use of the method, which has been applied to humans (Tenesa et al., 2007; McEvoy et al., 2011) as well as to several domesticated species (England et al., 2006; Uimari and Tapio, 2011; Corbin et al., 2012; Kijas et al., 2012). Along with these improvements, methodological limitations have become apparent and have been addressed here, with the majority of the efforts pointing to the correct estimation of recent *N_e_*. Yet, the quantitative value of the estimate is highly dependent on sample size, the type of LD estimation and the binning process (Waples and Do, 2008; Corbin et al., 2012), while its qualitative pattern depends more on the genetic information than on data manipulation.

So far this method has been applied using a variety of software, no standardized approach exists to bin the results and each study has applied a more or less arbitrary approach, e.g., binning for generation classes in the past (Corbin et al., 2012), binning for distance classes with a constant range for each bin (Kijas et al., 2012) or binning per distance classes in a linear fashion but with larger bins for the more recent time points (Burren et al., 2014). To our knowledge the only software available that estimate *N_e_* through LD is NeEstimator (Do et al., 2014), an upgraded version of the former LDNE (Waples and Do, 2008) allowing the analysis of large dataset (as 50k SNPChip). Importantly, while *SNeP* focuses on estimating historical *N_e_* trends, NeEstimator's aim is to produce contemporary unbiased *N_e_* estimates, the latter should therefore be considered as a complementary tool while investigating demography through LD.

We used *SNeP* to analyze two datasets where the method was previously applied. The results we obtained for the sheep data were both quantitatively and qualitatively comparable with those obtained by Burren et al. (2014), while for the Zebu data we obtained a *N_e_* trend estimate that closely matched that of Mbole-Kariuki et al. (2014) although our point estimates of *N_e_* were larger than those described for the data (Mbole-Kariuki et al., 2014). The discrepancy between these two results reflects that Burren and colleagues produced their *r*^2^ estimates using PLINK (the standard software for large scale SNP data manipulation) which uses the same approach used to estimate *r*^2^ by *SNeP*, while Mbole-Kariuki et al. followed Hao et al. (2007) for *r*^2^ estimation. The use of different estimates for LD is critical for the quantitative aspect of the *N_e_* curve, where due to the hyperbolic correlation between *N_e_* and *r*^2^, a decrease in *r*^2^ on its range closer to 0 can lead to a very large change in *N_e_* estimates, while differences in estimates are less significant when the *r*^2^ value is high, i.e., closer to 1. Therefore, although in one of the datasets the *N_e_* values where substantially different, in both cases the *N_e_* curves overlapped with those originally published.

As already suggested by other authors, the reliability of the quantitative estimates obtained with this method must be taken with caution, especially for *N_e_* values related to the most recent and the oldest generations (Corbin et al., 2012) because for recent generations, large values of *c* are involved, not fitting the theoretical implications that Hayes proposed to estimate a variable *N_e_* over time (Hayes et al., 2003). Estimates for the oldest generations might also be unreliable as coalescent theory shows that no SNP can be reliably sampled after 4*N_e_* generations in the past (Corbin et al., 2012). Further, *N_e_* estimates, and especially those related to generations further in the past, are strongly affected by data manipulation factors, such as the choice of MAF and alpha values. Additionally, the binning strategy applied can interfere with the general precision of the method, for example where an insufficient number of pairwise comparisons are used to populate each bin.

One of the applications of method is to compare breed demographies. In this case the shape of the *N_e_* curves would be the optimal tool to differentiate different demographic histories, more than their numerical values, by using them as a potential demographic fingerprint for that breed or species, yet taking into consideration that mutation, migration, and selection can influence the *N_e_* estimation through LD (Waples and Do, 2010). Additionally, careful consideration of the data analyzed with *SNeP* (and other software to estimate *N_e_*) is very important, as the presence of confounding factors such as admixture, may result in biased estimates of *N_e_* (Orozco-terWengel and Bruford, 2014).

The aim of *SNeP* is therefore to provide a fast and reliable tool to apply LD methods to estimate *N_e_* using high throughput genotypic data in a more consistent way. It allows two different *r*^2^ estimation approaches plus the option of using *r*^2^ estimates from external software. The use of *SNeP* does not overcome the limits of the method and the theory behind it, yet it allows the user to apply the theory using all corrections suggested to date.

## Author contributions

MB conceived and wrote the software and the manuscript. MB, MT, and POtW tested the software and performed the analyses. MT, POtW, and MWB revised the manuscript. All authors approved the final manuscript.

### Conflict of interest statement

The authors declare that the research was conducted in the absence of any commercial or financial relationships that could be construed as a potential conflict of interest.
